# Listen to Your Heart: Examining Modality Dominance Using Cross-Modal Oddball Tasks

**DOI:** 10.3389/fpsyg.2020.01643

**Published:** 2020-07-28

**Authors:** Christopher W. Robinson, Krysten R. Chadwick, Jessica L. Parker, Scott Sinnett

**Affiliations:** ^1^Department of Psychology, The Ohio State University at Newark, Newark, OH, United States; ^2^Occupational Therapy, The Ohio State University, Columbus, OH, United States; ^3^Department of Psychology, University of Tennessee, Knoxville, Knoxville, TN, United States; ^4^Department of Psychology, University of Hawai’i at Mānoa, Honolulu, HI, United States

**Keywords:** cross-modal processing, sensory dominance, attention, modality dominance, auditory processing, visual processing

## Abstract

The current study used cross-modal oddball tasks to examine cardiac and behavioral responses to changing auditory and visual information. When instructed to press the same button for auditory and visual oddballs, auditory dominance was found with cross-modal presentation slowing down visual response times more than auditory response times (Experiment 1). When instructed to make separate responses to auditory and visual oddballs, visual dominance was found with cross-modal presentation decreasing auditory discrimination, and participants also made more visual-based than auditory-based errors on cross-modal trials (Experiment 2). Experiment 3 increased task demands while requiring a single button press and found evidence of auditory dominance, suggesting that it is unlikely that increased task demands can account for the reversal in Experiment 2. Auditory processing speed was the best predictor of auditory dominance, with auditory dominance being stronger in participants who were slower at processing the sounds, whereas auditory and visual processing speed and baseline heart rate variability did not predict visual dominance. Examination of cardiac responses that were time-locked with stimulus onset showed cross-modal facilitation effects, with auditory and visual discrimination occurring earlier in the course of processing in the cross-modal condition than in the unimodal conditions. The current findings showing that response demand manipulations reversed modality dominance and that time-locked cardiac responses show cross-modal facilitation, not interference, suggest that auditory and visual dominance effects may both be occurring later in the course of processing, not from disrupted encoding.

## Introduction

Over the last 40 years, there has been a growing body of research examining how sensory systems process and integrate incoming information (Stein and Meredeth, 1993; Lewkowicz, 1994, 2000; Lickliter and Bahrick, 2000; Calvert et al., 2004; Driver and Spence, 2004; Spence, 2009, 2018; Robinson and Sloutsky, 2010a; Spence et al., 2012). Intersensory interactions can result in both facilitation and interference, and these effects appear to interact with the to-be-learned information. For example, speech perception, rhythm of a hammer tapping, identifying the location of car, and so on, can all be expressed in both the auditory and visual modalities, and numerous studies have shown that multisensory presentation can facilitate learning and speed up responses to redundant/amodal information compared to when the same information is presented to a single sensory modality (Miller, 1982; Giard and Peronnet, 1999; Bahrick and Lickliter, 2000; Bahrick et al., 2004; Molholm et al., 2004; Alsius et al., 2005; Colonius and Diederich, 2006; Sinnett et al., 2008).

However, in many situations, information presented to one sensory modality is irrelevant or may even conflict with information presented to a different sensory system. For example, phone conversations provide no information about upcoming traffic patterns, and streamed music is not related to the material written in a text, and so on. In situations where sensory modalities provide different or conflicting information, modality dominance effects can sometimes be observed with one modality attenuating processing in the other modality (Spence, 2009; Robinson and Sloutsky, 2010b; Spence et al., 2012). The primary goal of the current study was to examine possible mechanisms underlying modality dominance, and to address this goal, we used variations of change detection tasks (oddball procedures) to observe how cross-modal presentation and response demands affect auditory and visual discrimination. We also examined speed of discrimination by collecting both behavioral responses and time-locked psychophysiological changes (i.e., cardiac responses) as participants discriminated oddballs from standards.

Modality dominance effects are complex and fluctuate as a function of age, stimulus familiarity, response demands, nature of task, relative timing of multisensory stimuli, and signal strength (Welch and Warren, 1980; Sloutsky and Napolitano, 2003; Alias and Burr, 2004; Robinson and Sloutsky, 2004; Nava and Pavani, 2013; Robinson et al., 2016; Ciraolo et al., 2020). However, there is a growing body of research pointing to auditory dominance or increased reliance on auditory input early in development (Lewkowicz, 1988a,b; Sloutsky and Napolitano, 2003; Napolitano and Sloutsky, 2004; Robinson and Sloutsky, 2004, 2010c, 2019; Sloutsky and Robinson, 2008; Nava and Pavani, 2013; Hirst et al., 2018a, b). For example, some of these studies use variations of a change detection paradigm where infants or children are familiarized/habituated or briefly presented with an auditory–visual target item, which is followed by an auditory–visual test item. Infants and children often notice when the auditory component or when both auditory–visual components change (as indicated by increased looking or an explicit response); however, they often fail to notice when only the visual stimulus changes (Lewkowicz, 1988a,b; Sloutsky and Napolitano, 2003; Robinson and Sloutsky, 2004, 2010c; Sloutsky and Robinson, 2008). At the same time, infants and children ably discriminate pictures when presented in silence, suggesting that the auditory stimulus decreased the discriminability of the images (as indicated by infants not dishabituating to the changed visual stimuli or children failing to report that the visual stimuli changed). It is also important to note that pairing pictures and sounds together often had no cost on auditory processing. Thus, the signature pattern of auditory dominance in these change detection tasks is that cross-modal presentation attenuates visual processing while having little to no cost on auditory processing.

**FIGURE 1:**
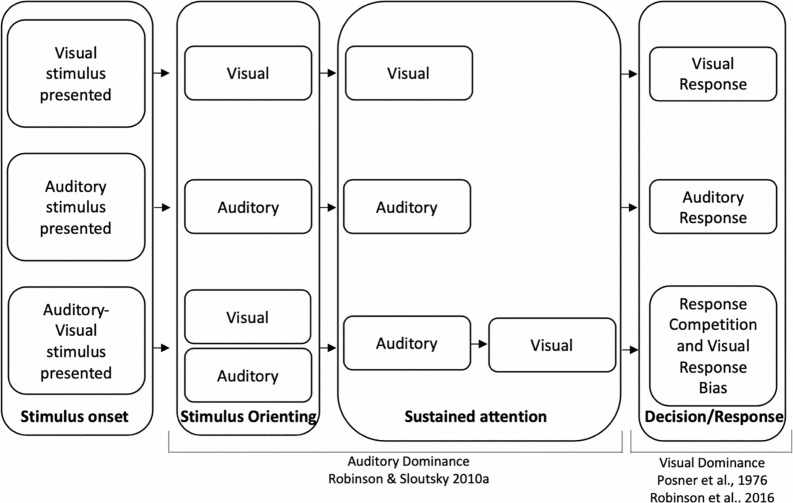
Timeline for unimodal visual processing (top), unimodal auditory processing (middle), and cross-modal processing (bottom). Auditory dominance occurs during sustained attention due to the serial nature of processing (Robinson and Sloutsky, 2010b) and visual dominance occurs because of a visual response bias, which may overshadow auditory dominance effects that occur during encoding (Posner et al., 1976; Robinson et al., 2016).

To account for auditory dominance effects, it has been suggested that sensory modalities compete for attentional resources (see also Wickens, 1984; Duncan et al., 1997; Eimer and Driver, 2000; Shimojo and Shams, 2001; Eimer and Van Velzen, 2002; Pavani et al., 2004; Sinnett et al., 2007; Robinson and Sloutsky, 2010b for related discussions on sensory competition). One potential mechanism underlying auditory dominance posits that the auditory modality should win the competition early in the course of processing (Robinson and Sloutsky, 2010b). The underlying idea is that auditory stimuli are often dynamic and transient in nature, and attention may automatically be deployed to these stimuli to ensure that they are processed before they disappear. Assuming that attention is a finite resource, automatically deploying attention to auditory input might come with a cost—attenuated or delayed visual processing. For example, when auditory and visual stimuli are presented unimodally (e.g., only sounds or pictures), stimulus detection and encoding of stimuli characteristics should occur early in the course of processing (see top two sections of Figure 1). However, when auditory and visual stimuli are presented simultaneously, the auditory stimulus may quickly engage attention, and the latency/duration of encoding the auditory stimulus should be comparable to the unimodal condition. Finally, because of the capacity limitations of attention, encoding the details of the visual stimulus may not begin until the auditory modality releases attention, which would result in a slowdown in visual processing relative to the unimodal visual baseline (see bottom section of Figure 1 relative to the unimodal visual baseline).

This conceptualization makes novel predictions regarding the dynamics of multisensory processing. For example, simultaneously presenting auditory and visual information should attenuate (or delay) visual processing, while having little to no cost on auditory processing (Robinson and Sloutsky, 2004, 2010c, 2019; Sloutsky and Robinson, 2008; Dunifon et al., 2016; Robinson et al., 2016; Barnhart et al., 2018; Robinson et al., 2018). The current approach also predicts that auditory stimuli that are slow to release attention (e.g., unfamiliar and/or complex stimuli) should attenuate or delay visual processing more than auditory stimuli that are quick to release attention (e.g., familiar and/or simple stimuli). Thus, giving infants exposure to the auditory stimulus prior to pairing it with a visual stimulus appears to attenuate auditory dominance effects possibly because they are processed more efficiently and are faster to release attention (Sloutsky and Robinson, 2008; Robinson and Sloutsky, 2010c). Finally, the account assumes that auditory dominance stems from auditory stimuli automatically engaging attention in a bottom-up manner because of the dynamic and transient nature of the input (exogenous attention), as opposed to auditory stimuli being prioritized as a strategic choice (endogenous attention). There are several studies supporting this claim. For example, children in Robinson and Sloutsky (2004) were presented with a sound and picture and required to use this information to predict where an animal would appear. In conditions where children showed evidence of auditory dominance, they often failed to learn that the visual cue predicted the animal’s location, even when children were consistently reminded to pay attention to the visual information (see Napolitano and Sloutsky, 2004; Dunifon et al., 2016 for a similar finding using an immediate recognition task where explicit instructions to pay attention to the pictures and ignore the sounds had little effect on reversing auditory dominance).

The competition for attention with transient auditory stimuli winning the competition account (Robinson and Sloutsky, 2010b) seems to capture some auditory dominance effects; however, it is difficult to reconcile these findings with approximately 40 years of visual dominance research showing that the visual modality typically dominates audition in adults (Sinnett et al., 2007; Spence, 2009; Spence et al., 2012). The most commonly used paradigm to study modality dominance in adults is the Colavita visual dominance task (Colavita, 1974; Colavita et al., 1976; Egeth and Sager, 1977; Colavita and Weisberg, 1979). In this task, participants are presented with auditory or visual information and are instructed to quickly respond by pressing one button when they hear an auditory stimulus and a different button when they see a visual stimulus. On a small percentage of trials, the auditory and visual stimuli are presented at the same time. Participants often erroneously respond to these cross-modal trials by pressing only the visual button, as opposed to pressing both buttons, or a third button associated with a cross-modal stimulus. Different stimulus manipulations (e.g., increasing auditory intensity levels, presenting auditory stimuli first, etc.) and implicit and explicit attentional manipulations directing attention to auditory stimuli sometimes weaken but fail to reverse visual dominance (Sinnett et al., 2007; Spence, 2009; Spence et al., 2012). For example, increasing the number of unimodal auditory stimuli (to increase attentional weights to auditory information) or explicitly asking participants to focus on the sounds (endogenous attention) appears to have little effect on reversing visual dominance.

Various accounts have been put forward to explain visual dominance in adults (Sinnett et al., 2007; Spence, 2009; Spence et al., 2012). One idea most directly related to the current study is that visual dominance might reflect a response bias directed toward the visual modality (endogenous attention) to compensate for the poor alerting qualities of the visual system (Posner et al., 1976). Accordingly, when visual and auditory stimuli are presented simultaneously, participants may strategically favor visual input to compensate for their low alerting properties, resulting in visual dominance. While visual dominance has been found using a variety of tasks, it is important to note that many of the studies pointing to visual dominance require participants to make speeded, modality-specific responses to auditory and visual input (Sinnett et al., 2007; Koppen et al., 2008; Sinnett et al., 2008; Ngo et al., 2010, 2011). Thus, it is unclear if visual stimuli are disrupting early processing (i.e., encoding of auditory input) or if interference happens later in the course of processing such as during the response/decision phase (see Spence, 2009 for a related discussion). It is possible to reconcile the discrepancy in modality dominance findings by positing that auditory stimuli are more likely to interfere during the encoding/detection phase and visual stimuli dominate during the decision/response phase.

Two recent studies provide preliminary support that different mechanisms may underlie auditory and visual dominance. Using a variation of an oddball paradigm, Ciraolo et al. (2020) and Robinson et al. (2016) presented adults with simple tones (unimodal auditory condition), monochromatic unfamiliar images (unimodal visual condition), or the tones and images paired together (cross-modal condition). Participants were repeatedly presented with the same tone, picture, or tone-picture pairing, were asked to inhibit responses to this stimulus (standard), and were asked to respond as quickly as possible if the tone, picture, or both tone and picture changed (auditory, visual, or cross-modal oddballs, respectively). Auditory dominance was observed when participants were required to press the same button for all oddball types with cross-modal presentation slowing down visual but not auditory processing (see Dunifon et al., 2016; Barnhart et al., 2018; Robinson et al., 2018 for a similar finding using an immediate recognition task). However, requiring participants to make separate responses to auditory, visual, and cross-modal oddballs reversed the effects, with cross-modal presentation having greater costs on auditory processing, which is consistent with visual dominance (Robinson et al., 2016). The pattern of modality dominance changed to visual dominance when we gave participants a chance to develop a modality-specific response bias in the three-button response condition; therefore, it was concluded that the different patterns of modality dominance may stem from different underlying mechanisms, with auditory stimuli disrupting the encoding of a visual stimulus and visual stimuli dominating the response (see Figure 1, which depicts auditory dominance occurring during sustained attention and visual dominance occurring during the decision/response phase).

The current study used variations of unimodal and cross-modal oddball procedures similar to the tasks reported in Robinson et al. (2016) and Ciraolo et al. (2020). Each participant in the three reported experiments was presented with unimodal auditory, unimodal visual, and cross-modal oddball tasks, and we compared the speed and accuracy of responding to oddballs when presented cross-modally with the respective unimodal baselines. If one modality dominates processing in another modality, then oddball detection in the losing modality should be slower and/or less accurate in the cross-modal condition compared to the unimodal condition (due to interference from the dominant modality). Thus, the signature pattern of modality dominance is reflected by asymmetric costs, with cross-modal presentation having a greater cost in the losing modality while having little or no cost on processing in the dominant modality. However, if slowdowns stem from increased task demands, then cross-modal presentation should equally attenuate processing in both modalities.

The primary goal of the current research was to better understand the possible mechanisms underlying modality dominance, and to achieve this goal, we have expanded on the study of Robinson et al. (2016) in several ways. First, given that many of the previous studies examining modality dominance used simple and/or unfamiliar auditory and visual stimuli (Colavita, 1974; Robinson et al., 2016; Ciraolo et al., 2020) we first wanted to make sure that effects could be generalized to more meaningful stimuli. For example, Robinson et al. (2016) used novel, monochromatic shapes and simple tones in a behavioral oddball task, and it is possible that these stimuli were integrated and perceived as a single percept. Given that semantic congruency appears to have no significant effect on sensory dominance when using a Colavita task (Koppen et al., 2008) it was hypothesized that the current study would replicate previous research and generalize to more meaningful stimuli where oddballs are not only less frequent, but they are also semantically incongruent. More specifically, when participants were asked to quickly make the same response to auditory and visual oddballs in Experiment 1, it was hypothesized that auditory dominance effects would be found. This would be consistent with the claim that auditory stimuli automatically engage attention and slow down or delay encoding of the visual stimulus. However, if visual stimuli dominate during the response/decision phase, then it should be possible to reverse modality dominance effects in Experiment 2 by requiring modality-specific responses (e.g., press one button for visual changes and a different button for auditory changes, etc.). Note that it is impossible to develop a visual response bias in Experiment 1 because participants are making the same response to auditory and visual oddballs.

The current study also expands on Robinson et al. (2016) by collecting cardiac data prior to the experiment proper and by examining real-time cardiac responses during the procedure. Whereas some psychophysiological variables were predictor variables [e.g., is it possible to predict modality dominance by knowing a person’s baseline heart rate variability (HRV)?], other variables were considered outcome variables (e.g., how quickly does the heart detect changes in auditory and visual information and how does cross-modal presentation affect these latencies?).

We looked at three predictor variables that might be related to modality dominance: unimodal auditory processing speed, unimodal visual processing speed, and baseline HRV. If modality dominance is a race between sensory modalities and the modality that is processed first dominates processing, then participants who are faster at processing sounds should be more likely to show auditory dominance, whereas participants who are faster at processing visual items (i.e., the pictures) should be more likely to show visual dominance. The proposed mechanism underlying auditory dominance makes a different prediction regarding processing speed, which posits that auditory stimuli automatically engage attention, thereby delaying processing of the details of a visual stimulus until the auditory modality releases attention (Robinson and Sloutsky, 2010b). If this is the case, then people who are faster at processing the sounds should start processing the visual information earlier in the course of processing (weak or no auditory dominance), and people who are slower at processing the sounds should show stronger auditory dominance because it takes longer for the auditory modality to release attention. At the same time, given the serial nature of processing during sustained attention (see Figure 1), visual processing speed should be less likely to predict modality dominance (i.e., Modality 1 delays processing of Modality 2, but Modality 2 has no effect on the already processed information in Modality 1). Finally, if modality dominance stems from a visual response bias or response competition, then the speed of encoding should not predict modality dominance.

It is also possible that HRV might also predict modality dominance. Heart rate variability research examines the complex interactions between the heart, brain, and behavior. The sinus node pacemaker controls the initiation of the heartbeat; however, the sympathetic nervous system and parasympathetic nervous system (via the vagal nerve) can both add variability to the beat by beat timing of the heart, with increased vagal activity slowing down the sinus node pacemaker (increasing time between beats) and vagal inhibition speeding up the sinus node pacemaker (decreasing time between beats). Moreover, individual differences in baseline HRV can be used as a proxy for vagal activity (Thayer and Lane, 2000) with increased HRV correlating with better selective attention (Park et al., 2013) emotional regulation (Williams et al., 2015) and response inhibition (Krypotos et al., 2011; Ottaviani et al., 2018). If modality dominance stems from endogenous attention directed to the visual modality (Posner et al., 1976) then it is possible that higher HRV might be associated with increased modality dominance. Alternatively, if better attentional control is associated with more efficient multisensory processing, then participants with higher HRV might be more likely to process information in both sensory modalities (weak or no modality dominance).

However, it is also possible that modality dominance effects stem from stimuli automatically engaging attention (exogenous attention) or from other lower-level mechanisms such as intersensory inhibition (Desimone and Duncan, 1995; Duncan, 1996; Spence et al., 2012). If this is the case, then HRV, a measure of top-down attentional control, should not predict modality dominance effects. This finding would be consistent with previous attentional manipulations, which have failed to reverse modality dominance in children and adults (Jennings, 1992; Napolitano and Sloutsky, 2004; Robinson and Sloutsky, 2004; Sinnett et al., 2007; Dunifon et al., 2016). Finally, while endogenous and exogenous attentional effects can occur at all stages of processing and responding, bottom-up effects such as stimulus saliency should be more pronounced in early stages of processing (e.g., pop-out effects, highly salient stimuli automatically engaging attention, etc.), whereas tasks to which participants are instructed to respond as quickly and as accurately as possible (i.e., focus on task-specific instructions) are more likely to rely on endogenous attention.

In regard to outcome variables, we also examined how quickly the heart responded to changing auditory and visual information (i.e., how quickly the cardiac response differed from pre-stimulus levels). In short, it was hypothesized that the cardiac responses to changing auditory and visual information would corroborate the behavioral data, especially if modality dominance effects stem from disrupted encoding. For example, if auditory stimuli disrupt/delay visual encoding, then auditory stimuli should slow down behavioral responses and slow down cardiac responses to changing visual information (i.e., cardiac responses to standards and oddballs should deviate earlier in the unimodal visual condition compared to the cross-modal condition). However, if cardiac responses are only sensitive to encoding and interference occurs in later stages of processing, then time-locked changes in heart rate (HR) to auditory and visual oddballs may not corroborate behavioral responses (i.e., visual oddballs are encoded at the same rate when presented unimodally or cross-modally).

While treating HR as an outcome variable is somewhat exploratory in nature, it is possible that time-locked cardiac responses may provide additional insight into the dynamics of cross-modal processing. For example, it is well documented that infants’ HRs slow down when actively encoding visual information (Richards and Casey, 1992) and using a modified oddball task, infants’ HRs also appear to slow down to novel, less frequent sounds than to more frequent sounds, suggesting longer periods of sustained attention to novel stimuli (Robinson and Sloutsky, 2010a). It is also well established that adult HRs slow down during stimulus orienting (Smith and Strawbridge, 1969) and on response inhibition tasks (Jennings et al., 1991, 1992; van der Veen et al., 2000; Hansen et al., 2003). Thus, it is possible that HR deceleration might also be associated with stimulus orienting, encoding, and response inhibition in adults. At the same time, HR should speed up when participants have to quickly make a response, and therefore, HR acceleration might also serve as a proxy for the timing associated with initiating the response. Thus, the HR analyses might shed light on the dynamics of cross-modal processing and provide a more detailed picture into the time course of cross-modal processing than behavioral measures such as response times and accuracies.

In summary, the current study employed three variations of an oddball task to examine cardiac responses and modality dominance effects. It was hypothesized that auditory stimuli would slow down visual oddball responses when auditory and visual oddball detections were associated with the same response (Experiments 1 and 3) and that this effect would stem from auditory input disrupting visual encoding, arguably due to attention being automatically engaged by auditory information. However, it was also hypothesized that participants should make more visual-based errors when auditory and visual responses were associated with different responses (Experiment 2), with cross-modal presentation attenuating auditory processing more than visual processing. We also examined if auditory processing speed, visual processing speed, and HRV could predict modality dominance effects, which could further highlight if these effects stem from endogenous and exogenous attention. Finally, cardiac responses were also collected throughout the experiment proper. If modality dominance stems from disrupted encoding, then cardiac responses to changing auditory and visual information may also be delayed in cross-modal conditions, although given the exploratory nature of this aspect of the study, it is hard to predict how this might map on to auditory or visual dominance effects.

## Experiment 1

Experiment 1 employed variations of unimodal and cross-modal oddball tasks requiring participants to make a single response to auditory and visual oddballs. Assuming that auditory input automatically engages attention and delays/disrupts visual processing (Robinson and Sloutsky, 2010b), it was hypothesized that cross-modal presentation would lead to a greater cost on visual processing relative to unimodal baselines (auditory dominance), and both response times and cardiac responses would show this delay in the cross-modal condition. Moreover, because auditory dominance does not appear to be under attentional control (Napolitano and Sloutsky, 2004; Robinson and Sloutsky, 2004; Dunifon et al., 2016) it was expected that baseline HRV would not predict which participants exhibit auditory dominance. Finally, assuming that the processing of the details of the visual item does not begin until the auditory modality releases attention, participants who are slower at processing auditory information should show stronger auditory dominance due to a longer dwell time of attention to auditory information.

### Methods

#### Participants

Thirty-eight adults (23 females, mean = 19.83 years, *SD* = 1.56 years) participated in Experiment 1. Sample size was based on behavioral research using variations of change detection paradigms (Dunifon et al., 2016; Robinson et al., 2016) where the average partial η^2^ for the predicted modality (auditory vs. visual) × presentation mode (unimodal vs. cross-modal) interaction is 0.26 (range = 0.18–0.36). Using G^∗^Power with a partial η^2^ of 0.26 and an α level of 0.05, it was estimated that 28 participants were needed to reach a power level of 0.9. It is important to note that effect sizes for the time-locked cardiac responses and HRV on cross-modal oddball tasks were not known; thus, we ran additional 10 participants to make sure we had sufficient power for the cardiac analyses. Participants were undergraduate students at The Ohio State University Newark who received course credit in exchange for participation. All participants in the final sample had normal or corrected to normal vision and hearing (self-reported) and provided consent prior to their participation. An additional six participants were tested but not included in the following analyses. Three participants were excluded because of self-reported hearing/vision loss or poor auditory/visual discrimination in the unimodal conditions, and three participants were excluded because of poor HR data (poor electrode placement, loose electrode, high number of artifacts, etc.).

#### Apparatus

A Dell Latitude E6430 laptop computer with DirectRT software was used for stimulus presentation and to record response times and accuracies. Visual stimuli were presented on a Dell P2212hB monitor, and auditory stimuli were presented via Kensington 33137 headphones at approximately 65 dB. A Dell Latitude E6430 laptop computer with Mindware software was used to record electrocardiograms. Two Ag–AgCl electrodes were placed on the participants’ right collarbone and left lower rib, and a reference electrode was placed on the participants’ right lower rib. Electrocardiograms were collected using a BioNex acquisition unit with a BioNex Impedance Cardiograph and GSC amplifier. DirectRT on the stimulus presentation laptop sent event markers to Bionex every time a stimulus was presented, thus time-locking electrocardiograms with the last stimulus presented on each trial (standard or oddball).

#### Materials

**FIGURE 2:**
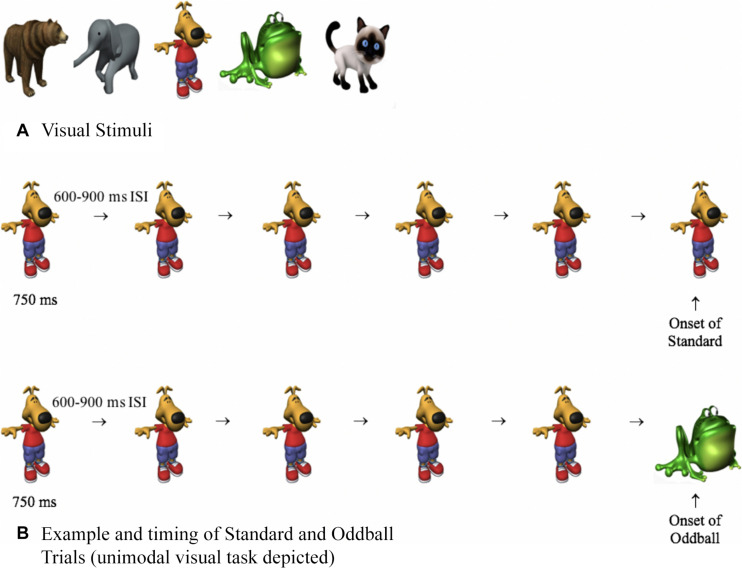
**(A)** Visual stimuli used in Experiments 1 to 3 and **(B)** examples and timing of standard and oddball trials. On both trial types, participants were presented with four or five standards. The last stimulus was either the standard (top of **B**) or an oddball (bottom of **B**), and response times and HR were time-locked with the onset of the last stimulus.

The stimulus pool consisted of five visual and five auditory stimuli. Visual stimuli (Figure 2) were approximately 400 × 400 pixels and pulsated centrally on a computer monitor for 750 ms, with a random 600- to 900-ms interstimulus interval (ISI). The timing between stimuli randomly varied, thus making it difficult to predict the timing of the next stimulus. The auditory stimuli consisted of bear, frog, elephant, cat, and dog sounds, which were taken from Marcell et al. (2000) and were shortened to 750 ms using Audacity software. As in basic oddball paradigms, one stimulus was frequently presented (approximately 90%, standard), and other stimuli were less frequent (approximately 10%, oddballs). The standard was a dog bark (unimodal auditory condition), an image of a dog (unimodal visual condition), or the dog and dog bark were paired together (cross-modal condition). The auditory and visual oddballs were an elephant, frog, cat, and bear, and the oddballs were created by only changing one aspect of the standard. For example, on auditory oddball trials, the picture of the dog (visual standard) may have been paired with a cat meow (auditory oddball), and on visual oddball trials, the dog bark (auditory standard) may have been paired with a picture of a cat (visual oddball). Thus, on oddball trials, the stimuli were not only less frequent, but they were also semantically incongruent (dog paired with meow).

#### Procedure

The study consisted of four phases. In the first phase, participants sat still for 5 min, while the computer recorded resting HRV. Participants then completed three different oddball tasks on the computer while their HR was monitored. The current study deviated from traditional oddball paradigms in that a trial was defined as a series of standards with either a standard or oddball at the end of the series (e.g., Trial 1: 5 standards → 1 oddball, Trial 2: 5 standards → 1 standard, etc.), as opposed to each stimulus being a trial. This manipulation gave the heart at least 6 s to respond to an oddball before encountering another oddball (assuming two short oddball sequences were presented back to back). The order of the three oddball tasks was randomized for each participant.

In the auditory oddball condition, there were 16 standard trials and 16 oddball trials. On auditory oddball trials, a dog bark was presented either four or five times, followed by one of the other animal sounds (oddball). On auditory standard trials, participants heard four or five dog barks, followed by another dog bark (standard). DirectRT sent an event marker to Bionex at the onset of the last standard or oddball in each trial. Participants in the unimodal auditory condition were instructed to look at a blank screen during the task. The unimodal visual condition was similar to the auditory condition, with the exception that the standard and oddballs were pictures, not sounds. Participants in the unimodal visual condition wore headphones, but no sounds were presented.

For each condition, we measured how quickly participants pressed the spacebar when they encountered an oddball and how quickly the heart differentiated standards and oddballs. Unlike traditional oddball procedures, there were equal numbers of standard trials (*n* = 16) and oddball trials (*n* = 16) in each condition. However, because the first four or five stimuli in each trial were standards, even on oddball sequences, 90% of the individual stimuli were standards (e.g., a standard trial with six stimuli and an oddball trial with six stimuli would have 11 standards and one oddball, or 92% standards).

In the cross-modal condition, the trials consisted of four or five standard image-sound pairs (picture of dog paired with dog bark) followed by another image-sound pair that was either a standard or an oddball. Each participant had a total of 96 cross-modal trials (48 standard and 48 oddballs). Sixteen of the oddball trials were visual oddballs, and 16 were auditory oddballs. On visual oddball trials, the dog bark (auditory standard) was paired with one of the four visual oddballs (e.g., cat). On auditory oddballs, the picture of the dog (visual standard) was paired with one of the four auditory oddballs (e.g., meow). Lastly, there were also 16 double oddball trials, where both auditory and visual stimuli changed (e.g., picture of cat paired with meow). Both components of double oddballs were always new but congruent (e.g., cat paired with meow, frog paired with rabbit, etc.). As in the unimodal conditions, each stimulus was presented for 750 milliseconds with a random 600- to 900-ms ISI, and the auditory and visual stimuli had the same onset and offset. See Table 1 for all trial types and frequencies. Unimodal auditory trials, unimodal visual trials, and cross-modal trials were blocked, and the order of blocks and trials within each block (e.g., auditory oddball, double oddball, etc.) were randomized for each participant.

**TABLE 1:** Stimulus structure of three oddball tasks in Experiments 1–3 (frequency of each stimulus).

	**Unimodal Auditory**	**Unimodal Visual**	**Cross-modal**
Standard	A1 (*N* = 16)	V1 (*N* = 16)	A1V1 (48)
Auditory Oddballs	A2 (*N* = 4)		A2V1 (*N* = 4)
	A3 (*N* = 4)		A3V1 (*N* = 4)
	A4 (*N* = 4)		A4V1 (*N* = 4)
	A5 (*N* = 4)		A5V1 (*N* = 4)
Visual Oddballs		V2 (*N* = 4)	A1V2 (*N* = 4)
		V3 (*N* = 4)	A1V3 (*N* = 4)
		V4 (*N* = 4)	A1V4 (*N* = 4)
		V5 (*N* = 4)	A1V5 (*N* = 4)
Double Oddballs			A2V2 (*N* = 4)
			A3V3 (*N* = 4)
			A4V4 (*N* = 4)
			A5V5 (*N* = 4)

*A, auditory; V, visual; AV, audiovisual. The values denote individual stimuli (e.g., V1–V5 are associated with the dog, bear, cat, elephant, and frog images, respectively).*

### Results and Discussion

We begin by focusing on the behavioral data, followed by time-locked cardiac responses and predictor variable analyses.

#### Behavioral Analyses

In regard to the 16 standard trials, participants only false alarmed on 0.40% of the trials; thus, we focused on the proportion of hits to oddballs in the primary analyses below. Each oddball trial was classified as correct if the participant pressed spacebar when presented with an oddball or incorrect if the participant did not press the spacebar within 1,500 ms after the onset of the oddball. Preliminary analyses examined the saliency and discriminability of the different oddballs when presented unimodally (bear, frog, etc.). Proportion of hits on unimodal trials were submitted to a 2 (modality: auditory vs. visual) × 4 (oddball: bear, cat, elephant, frog) repeated-measures analysis of variance (ANOVA). The analysis revealed no effects or interactions, *p*’s > 0.34; thus, we averaged across the different oddballs in the primary analyses.

**FIGURE 3:**
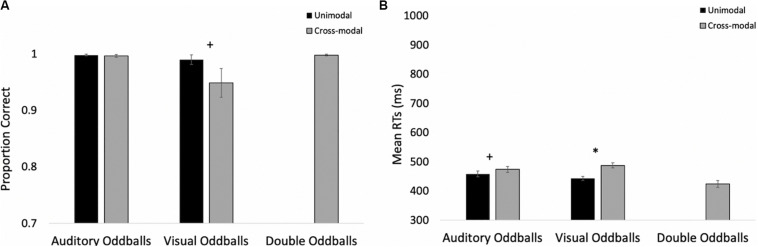
Proportion of correct responses **(A)** and mean response times **(B)** across trial types and conditions in Experiment 1. Error bars denote standard errors, and “+” and “*” denote cross-modal means differ from unimodal means, *p*’s < 0.05 and 0.001, respectively.

The proportions of hits on auditory and visual oddballs were submitted to a 2 (modality: auditory vs. visual) × 2 (presentation mode: unimodal vs. cross-modal) repeated-measures ANOVA (see Figure 3A for means and standard errors). The analysis revealed an effect of presentation mode, *F*(1,37) = 4.63, *p* = 0.038, *η*_p_^2^ = 0.11, BF10 = 0.67, with proportion correct being higher in the unimodal conditions (mean = 0.99, *SE* = 0.01) than in the cross-modal conditions (mean = 0.97, *SE* = 0.01). The effect of presentation mode was corroborated with a Wilcoxon signed ranks test, *Z* = −2.91, *p* = 0.004, which is a non-parametric repeated-measures test that does not assume normality. The effect of modality and the modality × presentation mode interaction failed to reach statistical significance, *p*’s > 0.09.

We also examined response times on auditory and visual oddball trials. To determine if there were any differences in saliency/discrimination across the different oddballs, we submitted unimodal response times to a 2 (modality: auditory vs. visual) × 4 (oddball: bear, cat, elephant, frog) repeated-measures ANOVA. While participants were faster at responding to visual stimuli (mean = 458 ms, *SE* = 9.91) than the auditory stimuli (mean = 443 ms, *SE* = 6.95), *F*(1,37) = 4.13, *p* = 0.049, *η*_p_^2^ = 0.10, BF10 = 12.29, there were no differences in response times across any of the oddballs, and response times to the different oddballs did not interact with stimulus modality, *p*’s > 0.27; therefore, we averaged across the oddball types in the primary analyses.

Primary response time analyses examined response times on correct trials in the cross-modal condition when only the auditory or visual component changed. We compared these response times to the respective unimodal baselines (see Figure 3B for means and standard errors). Because response times are typically skewed, we report mean response times in all of the figures, but used log-transformed response times in all analyses. Log-transformed response times on correct trials were submitted to a 2 (modality: auditory vs. visual) × 2 (presentation mode: unimodal vs. cross-modal) repeated-measures ANOVA. The analysis revealed an effect of presentation mode, *F*(1,37) = 35.71, *p* < 0.001, *η*_p_^2^ = 0.49, BF10 = 9.14 × 10^5^, and a modality × presentation mode interaction, *F*(1,37) = 9.89, *p* = 0.003, *η*_p_^2^ = 0.21, BF10 = 1.83 × 10^5^. As can be seen in Figure 3B, compared to the unimodal baselines, cross-modal presentation slowed visual responses (45-ms slowdown) more than auditory responses (15-ms slowdown). The effect of modality did not reach significance, *p* = 0.63.

The response time data show that cross-modal presentation attenuated visual processing more than auditory processing, a finding consistent with auditory dominance. However, it is important to note that there was no decrease in accuracy or a slowdown in response times when both modalities changed (see Figures 3A,B, respectively). In fact, response times on these trials were faster than all trial types, *t*’s > 4.82, *p*’s < 0.001, BF10’s > 877, suggesting that the slowdown on visual oddball trials occurs because of the conflicting information (e.g., auditory standard elicits no response; whereas, visual oddball elicits button press), as opposed to any auditory stimulus slowing down processing and/or cross-modal presentation increasing task demands.

#### Time-Locked HR Analyses

We also examined real-time cardiac responses time-locked with the final stimulus in the sequence (either a standard or oddball). The primary goal of this analysis was to use a psychophysiological measure to determine the onset of discrimination (point where the heart responds differently to standards and oddballs) and to determine if the onset of visual discrimination (HR acceleration/deceleration) occurs later in the course of processing in the cross-modal condition. Mindware software was used for artifact detection/correction and to export data. Weighted interbeat intervals (IBIs) were exported every second, and difference waveforms were calculated by subtracting pre-stimulus IBI from each 1-s IBI bin post-stimulus. Note that IBIs reflect the time between heartbeats; thus, increases in IBI reflect slowed HR, and difference IBIs greater than 0 reflect slowed HRs compared to pre-stimulus levels, whereas values less than 0 reflect faster HRs compared to pre-stimulus levels. Paired *t* tests comparing standard and oddball IBIs were conducted each second to determine how quickly the heart differentiated oddballs from standards.

**FIGURE 4:**
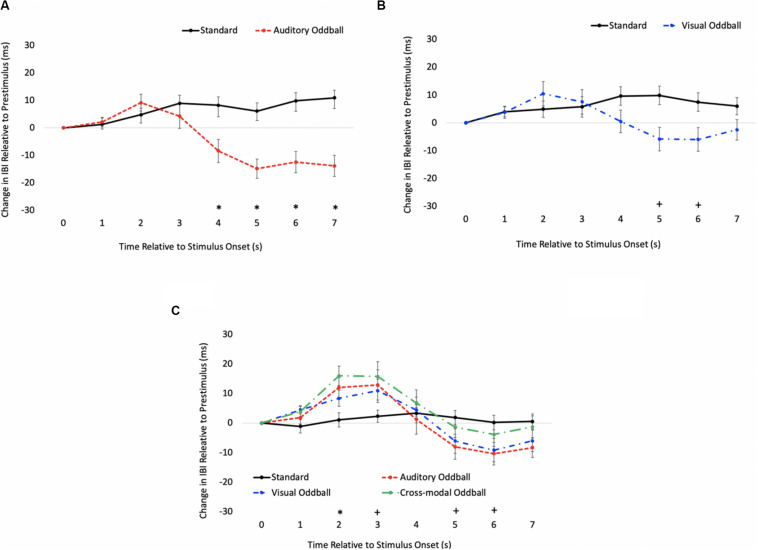
Cardiac responses in Experiment 1 across time in unimodal auditory **(A)**, unimodal visual **(B)**, and cross-modal conditions **(C)**. Error Bars denote standard errors. In panels **(A,B)**, “+” and “*” denote that oddballs differ from standard, *p*’s < 0.05 and 0.001, respectively. In panel **(C)**, “+” and “*” denote that visual oddball (auditory standard/visual oddball) differs from standard (auditory standard/visual standard), *p*’s < 0.05 and 0.001.

The HR waveforms for unimodal auditory, unimodal visual, and cross-modal conditions are presented in Figures 4A–C, respectively. As can be seen in Figures 4A,B, cardiac responses to oddballs and standards differed at 4 s after stimulus onset in the auditory condition and 5 s after stimulus onset in the visual condition. Note that these effects were primarily driven by HR acceleration to oddballs; contrary to what is observed in infant tasks, where infants show slower HR to oddballs (Robinson and Sloutsky, 2010a). Thus, the acceleration in adults likely stems from participants making a speeded response to oddballs and making no response to standards. However, discrimination of visual oddballs occurred earlier in the cross-modal condition (2 s after stimulus onset) compared to the 5 s in the unimodal visual condition. Thus, the behavioral data point to cross-modal interference with cross-modal presentation slowing down visual response times, but changes in time-locked cardiac responses show facilitation, with discrimination occurring earlier in the course of processing when information is presented to both sensory modalities.

#### Predictors of Modality Dominance

To examine the relationship between HRV and modality dominance, we calculated a measure of resting HRV for each participant during the 5-min baseline phase. Mindware software was used to isolate the baseline phase and for artifact detection, and root mean square of the successive differences (RMSSD) was calculated for each participant, with RMSSD quantifying HRV. Higher RMSSD values indicate more variability in resting HR (i.e., better attentional control). If modality dominance stems from participants with better attentional control using endogenous attention to focus on one modality more than another, then RMSSD should be positively correlated with modality dominance, whereas if participants with better top-down control of attention are more efficient at processing multisensory stimuli, then participants with higher RMSSD should be more likely to encode both modalities and be less likely to show modality dominance.

We were also interested if auditory and visual processing speed could account for modality dominance effects. For example, if modality dominance is a race between sensory modalities, then participants who are faster at processing the visual information should show visual dominance, and participants who are faster at processing the auditory information should show auditory dominance. However, it is also possible that auditory stimuli automatically engage attention, and visual processing does not begin until the auditory modality releases attention (Robinson and Sloutsky, 2010b). According to this account, auditory processing speed should be a better predictor of auditory dominance with participants who are slower at processing the sounds showing stronger auditory dominance, given that theoretically it would take longer for the auditory modality to release attention.

Heart rate variability (RMSSD), unimodal auditory response times, and unimodal visual response times served as predictor variables in a stepwise regression to determine which variables account for modality dominance. Recall that the signature pattern of modality dominance is that cross-modal presentation attenuates processing in one modality more than the other. To quantify modality dominance, we first calculated costs of cross-modal presentation separately for auditory and visual modalities (cross-modal response times – unimodal response times), with values greater than zero indicating that cross-modal presentation slowed response times^1^. We then calculated a difference score for each participant by subtracting auditory cost from visual cost (cost of cross-modal presentation on visual processing – cost of cross-modal presentation on auditory processing). Values greater than zero indicate that cross-modal presentation had a greater cost on visual processing (auditory dominance), and scores less than zero indicated that cross-modal presentation had a greater cost on auditory processing (visual dominance).

**FIGURE 5:**
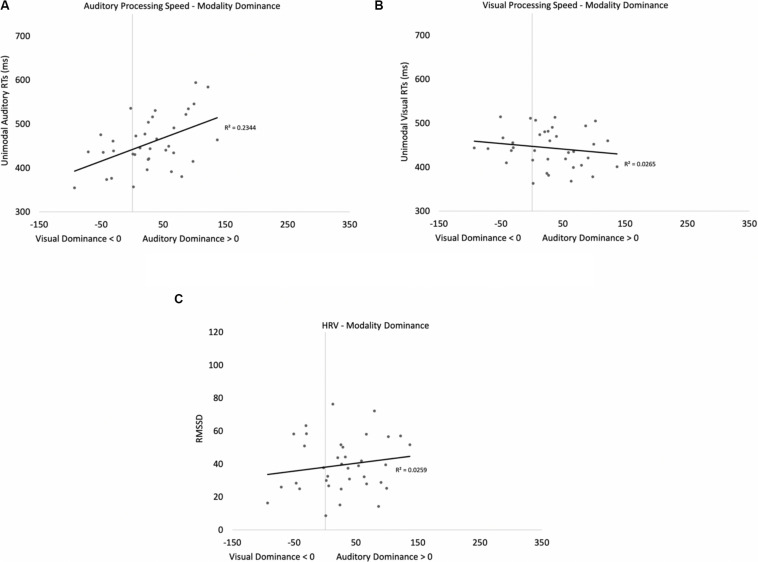
Scatterplots and effect sizes for unimodal auditory processing **(A)**, unimodal visual processing **(B)**, and HRV **(C)** with modality dominance (cost of cross-modal presentation on visual – cost of cross-modal presentation on auditory) in Experiment 1. Values greater than zero on the *x*-axis are consistent with auditory dominance.

Two participants were removed from the following analysis because their data were greater than two standard deviations above the mean. The bivariate scatterplots and effect sizes for the three predictor variables on the dependent variable are presented in Figure 5. Seventy-five percent of the participants exhibited greater costs on visual processing (auditory dominance). While auditory processing speed accounted for 23% of the variance in modality dominance (slower auditory processing was associated with stronger auditory dominance), visual processing speed and HRV each accounted for only 3% of the variance.

Auditory processing speed, visual processing speed, and HRV were also entered into a stepwise regression, which yielded two significant models. In the first model, auditory processing (*b* = 0.48) significantly predicted modality dominance, *F*(1,34) = 10.41, *p* = 0.003, *R*^2^ = 0.21, BF10 = 13.65. The same pattern emerges when using relative processing speed as a predictor variable (unimodal auditory RT – unimodal visual RT), with slower auditory processing relative to visual also being associated with auditory dominance. In the second model, auditory processing speed (*b* = 1.05, *p* < 0.001) and visual processing speed (*b* = −0.86, *p* < 0.001) both predicted modality dominance, *F*(2,33) = 30.31, *p* < 0.001, *R*^2^ = 0.65, BF10 = 3.69 × 10^5^, with auditory dominance being more pronounced in participants who were slower at processing the sounds or faster at processing the pictures. However, caution is required when interpreting the unimodal visual response time data given its correlation/multicollinearity with auditory response times, *r*(34) = 0.66, *p* < 0.001. Heart rate variability was not correlated with auditory or visual processing speeds (*p*’s > 0.56), and HRV did not significantly account for variability in modality dominance effects, *p* = 0.35.

In summary, while response time data point to auditory dominance, time-locked changes in HR show faster visual discrimination in the cross-modal condition than the unimodal visual condition (cross-modal facilitation). Changes in HR to standards and oddballs likely stem from participants only responding to oddballs; however, all reported experiments compared cardiac responses to oddballs in the unimodal condition (where participants made a response) with cardiac responses to the same oddballs when presented cross-modally (where participants also made a response). Thus, differences between unimodal and cross-modal conditions cannot stem from the response. Finally, while HRV did not predict auditory dominance, unimodal auditory processing speed best accounted for auditory dominance, with stronger auditory dominance being associated with slower auditory processing.

## Experiment 2

While Experiment 1 provides behavioral support for the claim that auditory stimuli may disrupt or delay visual processing (Robinson and Sloutsky, 2010c), it is difficult to reconcile these findings with approximately 40 years of research pointing to visual dominance in young adults (Sinnett et al., 2007; Spence, 2009; Spence et al., 2012). Moreover, given the numerous differences between the cross-modal oddball task reported in Experiment 1 and variations of the Colavita (1974) visual dominance, it is unclear what factors best account for the discrepancy in findings. Experiment 2 examines one factor that may account for modality dominance and follows up on the idea that auditory and visual dominance may stem from different underlying mechanisms, with auditory stimuli engaging attention early and disrupting visual encoding, and visual stimuli winning the competition during the decision/response phase (Robinson et al., 2016). If visual dominance stems from a response bias to compensate for visual stimuli being less likely to engage attention (Posner et al., 1976), then participants should show visual dominance if given an opportunity to develop a visual response bias.

Experiment 2 tested this hypothesis by using the same procedure and stimuli as reported in Experiment 1; however, participants in Experiment 2 also had to report if they encountered an auditory oddball, visual oddball, or double oddball using separate key responses. It was hypothesized that requiring separate responses to auditory, visual, and double oddballs would give participants the opportunity to develop a visual response bias, which, in turn, should reverse the pattern of dominance from auditory to visual. If these effects stem from top-down control of attention (Posner et al., 1976) then it is possible that HRV will predict which participants show visual dominance effects.

### Methods

#### Participants, Materials, and Procedure

Forty-two new participants (23 females, mean = 20.14 years, *SD* = 4.00 years) from The Ohio State University Newark participated in Experiment 2. An additional six participants were tested but not included in the following analyses. One participant was excluded because of poor auditory discrimination in the unimodal condition; three participants were excluded because they forgot which buttons were associated with the different oddballs in the cross-modal condition, and two participants were excluded because of poor HR data. The stimuli and procedure were identical to Experiment 1 except that participants were instructed to press 1 on the number pad if the auditory component changed, 2 if the visual component changed, and 3 if both modalities changed. Button-modality pairings were counterbalanced across subjects. In the unimodal condition, participants were instructed to press only one of the buttons.

### Results and Discussion

As in Experiment 1, we first present the behavioral data followed by the HR and predictor variable analyses.

#### Behavioral Analyses

Participants only false alarmed on 0.26% of the standard trials; thus, primary analyses focused exclusively on oddball trials. Preliminary analyses examined the saliency and discriminability of the different oddballs when presented unimodally (bear, frog, etc.). Proportion of hits on unimodal trials were submitted to a 2 (modality: auditory vs. visual) × 4 (oddball: bear, cat, elephant, frog) repeated-measures ANOVA. None of the effects reached significance, and the main effect of oddball and oddball by modality interaction did not reach significance, *p*’s > 0.27; thus, we averaged across the oddball types.

The proportions of correct responses on auditory and visual oddballs were submitted to a 2 (modality: auditory vs. visual) × 2 (presentation mode: unimodal vs. cross-modal) repeated-measures ANOVA. The analyses revealed an effect of modality, *F*(1,41) = 9.81, *p* = 0.003, *η*_p_^2^ = 0.19, BF10 = 8.63, with more accurate responding to visual oddballs (mean = 0.96, *SE* = 0.01) than auditory oddballs (mean = 0.90, *SE* = 0.01). The analysis also revealed an effect of presentation mode, *F*(1,41) = 82.46, *p* < 0.001, *η*_p_^2^ = 0.67, BF10 = 9.84 × 10^9^, with more accurate responding on unimodal trials (mean = 0.99, *SE* = 0.01) than cross-modal trials (mean = 0.86, *SE* < 0.01). Both of the main effects were corroborated with a Wilcoxon signed ranks test, *Z*’s > −2.84, *p*’s < 0.004.

**FIGURE 6:**
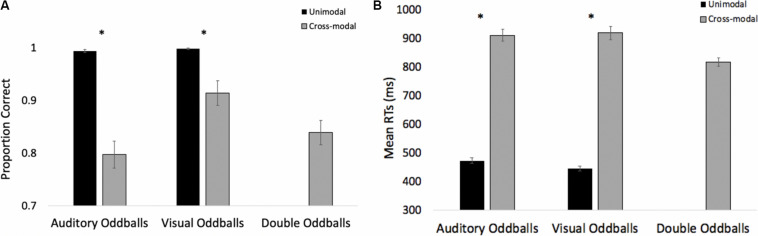
Proportion of correct responses **(A)** and mean response times **(B)** across trial types and conditions in Experiment 2. Error bars denote standard errors, and “*” denotes cross-modal means differ from unimodal means, *p*’s < 0.001.

The analysis also revealed a modality × presentation mode interaction, *F*(1,41) = 8.44, *p* = 0.006, *η*_p_^2^ = 0.17, BF10 = 3.60 × 10^11^. As can be seen in Figure 6A, cross-modal presentation attenuated accuracy in both modalities. However, compared to the unimodal baselines, accuracy dropped by 20% in the auditory modality, whereas accuracy dropped by only 8% in the visual modality. Note that the proportions of correct auditory and visual oddball detections in the current experiment were in the opposite direction compared to Experiment 1, with the cost of cross-modal presentation being greater for auditory oddball detection than visual oddball detection.

To explore Colavita visual dominance effects, we examined errors made on double oddballs. Recall that a correct response on double oddball trials is to press the button associated with a cross-modal stimulus. Pooled across participants, the overall error rate to double oddballs was 16.1%. Of the 108 errors made, there were 12 misses where participants failed to make any response. On the remaining error trials, participants pressed the visual button 69 times, and the auditory button 27 times, resulting in a visual modality bias, *χ^2^* (1, *n* = 96) = 18.38, *p* < 0.001.

Additional analyses focused on response times on correct trials. To determine if there were any differences in saliency/discrimination across the different oddballs, we submitted unimodal response times to a 2 (modality: auditory vs. visual) × 4 (oddball: bear, cat, elephant, frog) repeated-measures ANOVA. While participants were faster at responding to visual oddballs (mean = 447 ms, *SE* = 8.83) than auditory oddballs (mean = 474 ms, *SE* = 10.20), *F*(1,41) = 7.49, *p* = 0.009, *η*_p_^2^ = 0.16, BF10 = 2817.15, the main effect of oddball and the oddball × modality interaction did not reach significance, *p*’s > 0.14; therefore, we averaged across the oddball types.

Response times across modality and presentation mode are presented in Figure 6B. Log-transformed response times were submitted to a 2 (modality: auditory vs. visual) × 2 (presentation mode: unimodal vs. cross-modal) repeated-measures ANOVA. The analysis revealed an effect of presentation mode, *F*(1,41) = 963.19, *p* < 0.001, *η*_p_^2^ = 0.96, BF10 = 5.63 × 10^72^, with participants responding approximately 470 ms faster in the unimodal conditions than in the cross-modal conditions. The effect of modality and the modality × presentation mode interaction failed to reach statistical significance, *p*’s > 0.10.

#### Time-Locked HR Analyses

**FIGURE 7:**
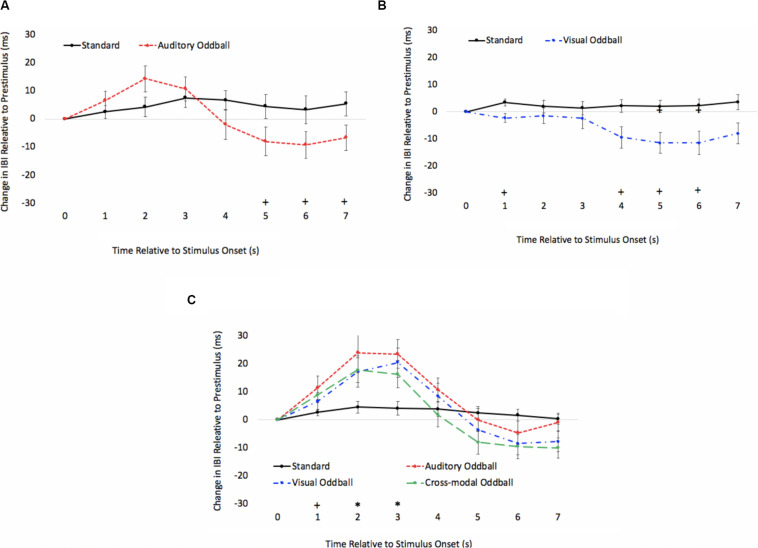
Cardiac responses in Experiment 2 across time in unimodal auditory **(A)**, unimodal visual **(B)**, and cross-modal conditions **(C)**. Error bars denote standard errors. In panels **(A,B)**, “+” and “*” denote that oddballs differ from standard, *p*’s < 0.05 and 0.001, respectively. In panel **(C)**, “+” and “*” denote that auditory oddball (auditory oddball/visual standard) differs from standard (auditory standard/visual standard).

As in Experiment 1, we examined real-time cardiac responses as participants were presented with standards and oddballs, and Mindware was used for artifact detection/correction. The HR waveforms for unimodal auditory, unimodal visual, and cross-modal conditions are presented in Figures 7A–C, respectively. As can be seen in Figures 7A,B, there was some evidence that participants discriminated auditory stimuli 5 s after stimulus onset and discriminated visual stimuli 1 s after stimulus onset. However, as can be seen in Figure 7C, participants discriminated auditory stimuli 1 s after stimulus onset in the cross-modal condition, which was faster than the unimodal auditory baseline. Thus, while accuracy and errors made on cross-modal oddball trials both point to visual dominance, HR analyses point to cross-modal facilitation with faster discrimination in the cross-modal condition.

#### Predictors of Modality Dominance

**FIGURE 8:**
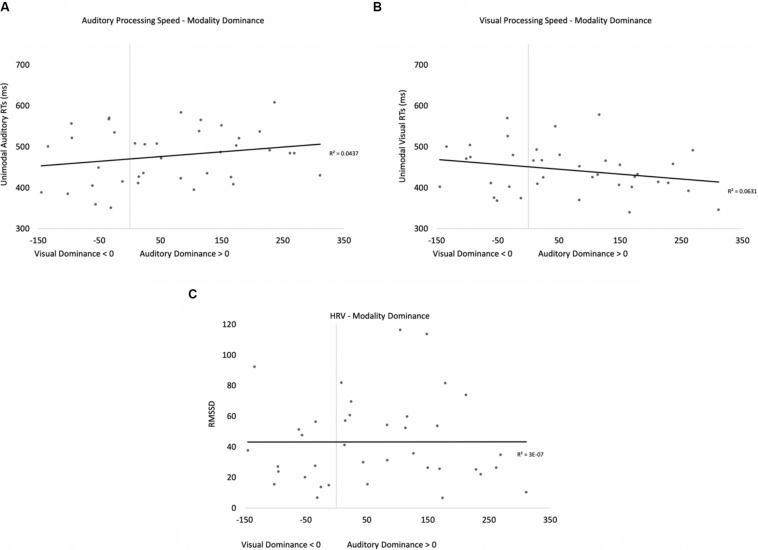
Scatterplots and effect sizes for unimodal auditory processing **(A)**, unimodal visual processing **(B)**, and HRV **(C)** with modality dominance (cost of cross-modal presentation on visual – cost of cross-modal presentation on auditory) in Experiment 2. Values greater than zero on the *x* axis are consistent with auditory dominance.

As in Experiment 1, we examined if HRV and processing speed could account for modality dominance. Four participants were removed because their data were greater than two standard deviations above the mean. See Figure 8 for the bivariate scatterplots and effect sizes for the three predictor variables. Sixty-two percent of the participants exhibited greater costs on visual processing (auditory dominance). Heart rate variability (RMSSD), unimodal auditory response times, and unimodal visual response times served as predictor variables in a stepwise regression to determine which variables account for modality dominance (cost of cross-modal presentation on visual processing - cost of cross-modal presentation on auditory processing). As in Experiment 1, unimodal auditory and unimodal visual response times were correlated, *r*(38) = 0.56, *p* < 0.001. However, in contrast to Experiment 1, auditory processing speed accounted for only 4% of the variance in modality dominance, and none of the variables significantly predicted modality dominance in Experiment 2. Heart rate variability was not correlated with auditory or visual processing speeds (*p*’s > 0.87), and it did not significantly account for variability in modality dominance effects, *p* = 0.99. We also examined if HRV and processing speed could account for more traditional measures of modality dominance. There was also no evidence that unimodal auditory processing speed, unimodal visual processing speed, or HRV correlated with the number of visual-based errors made on double oddballs, *p*’s > 0.21.

### Across Experiment Comparisons

Although we only changed response demands in Experiment 2, it could be argued that there is a confounding across experiments because we also increased cognitive load. Support for this claim comes from the finding that cross-modal presentation slowed down responding more in Experiment 2 (mean = 474 ms, *SE* = 2.46) than in Experiment 1 (mean = 33 ms, SE = 1.58), *t*(78) = 146.98, *p* < 0.001, BF10 = 4.06 × 10^92^. Cross-modal presentation also resulted in more errors in Experiment 2 (mean = 0.13, *SE* < 0.01) than in Experiment 1 (mean = 0.03, *SE* < 0.01), *t*(78) = 14.76, *p* < 0.001, BF10 = 1.59 × 10^21^. The goal of Experiment 3 was to address the potential confounding across Experiments 1 and 2.

## Experiment 3

The behavioral data from Experiments 1 and 2 point to auditory dominance when participants had to make the same response to auditory and visual oddballs (Experiment 1) and visual dominance when participants had to make different responses to auditory and visual oddballs (Experiment 2). These findings are consistent with the claim that auditory stimuli automatically engage attention and delay/disrupt visual encoding (Robinson and Sloutsky, 2010b) and that visual dominance may stem from a response bias to compensate for the poor alerting properties of visual stimuli (Posner et al., 1976). Recall that in Experiment 1 it was impossible to develop a modality-specific response bias because auditory and visual discrimination was associated with the same response. When removing a potential mechanism that may underlie visual dominance, visual dominance disappeared in Experiment 1, and auditory stimuli slowed down visual processing.

However, given the increased response times and decreased accuracies in Experiment 2, it is unclear if the shift from auditory-to-visual dominance stems from increased cognitive load or from requiring participants to make separate responses to auditory and visual information. Although at face value it may seem difficult to disentangle cognitive load from increased response options, the goal of Experiment 3 was to increase cognitive load, while requiring participants to make the same response to auditory and visual oddballs. Experiment 3 addressed this issue by making a small change to Experiment 1. As in Experiment 1, participants were instructed to make the same response if the auditory or visual modality changed. However, in contrast to Experiment 1, participants were instructed to withhold responses when both modalities changed (i.e., double oddballs, see also Robinson et al. (2016) and Ciraolo et al. (2020), for a similar manipulation of cognitive load). Effectively, this manipulation increases cognitive load because participants are no longer able to make a response based on whether they detect a change in a single modality. Rather, they are now required to check the other modality to determine if they should make a response (auditory and visual oddballs) or inhibit their response (double oddballs).

If the reversal to visual dominance observed in Experiment 2 is driven solely by the use of separate responses for auditory and visual input (i.e., potentially leading to a response bias), then auditory dominance should be found in Experiment 3 because participants are no longer able to develop a visual response bias given that they are making the same response to both oddball types. However, if increasing task demands are driving the reversal to visual dominance, then visual dominance should also be found in the current experiment because participants have to consider both modalities before responding to auditory and visual oddballs and therefore periodically inhibit responses—a task that is more difficult than Experiment 2.

### Methods

#### Participants, Materials, and Procedure

Twenty-eight participants (15 females, mean = 19.50 years, *SD* = 0.86 years) from The Ohio State University Newark participated in Experiment 3. An additional three participants were tested but not included in the following analyses. One participant was excluded because of poor auditory discrimination in the unimodal condition, and two participants were excluded because of poor HR data. With the following exception, the stimuli, design, and procedure were identical to Experiment 1. In the current experiment, participants had to inhibit their response when presented with a double oddball (when both modalities changed).

## Results and Discussion

### Behavioral Analyses

As in previous experiments, participants made very few errors on standard trials (1.34% false alarm rate), so we focused only on the proportion of hits to oddballs. To determine if there were any differences in saliency/discrimination across the different oddballs, we submitted the proportion of hits to a 2 (modality: auditory vs. visual) × 4 (oddball: bear, cat, elephant, frog) repeated-measures ANOVA. There were no differences in accuracy across any of the oddballs, and oddball did not interact with stimulus modality, *p*’s > 0.09; thus, we averaged across the oddball types.

**FIGURE 9:**
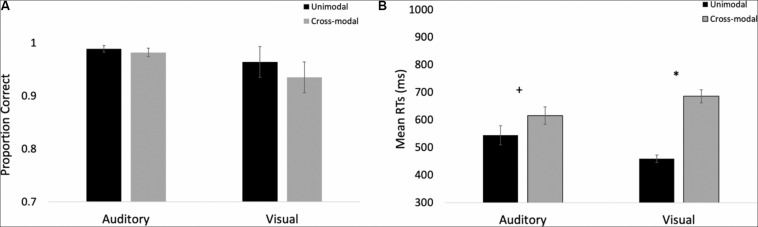
Proportion of correct responses **(A)** and mean response times **(B)** across trial types and conditions in Experiment 3. Error bars denote standard errors, and “+” and “*” denote cross-modal means differ from unimodal means, *p*’s < 0.05 and 0.001, respectively.

The proportions correct on auditory and visual oddball trials were submitted to a 2 (modality: auditory vs. visual) × 2 (presentation mode: unimodal vs. cross-modal) repeated-measures ANOVA (see Figure 9A for means and standard errors). The analysis revealed an effect of modality, *F*(1,27) = 4.38, *p* = 0.046, *η*_p_^2^ = 0.14, BF10 = 0.99, with the proportion of correct responses on auditory trials (mean = 0.99, *SE* < 0.01) being significantly greater than on visual trials (mean = 0.95, *SE* = 0.02). The effect of presentation mode and the modality × presentation mode interaction failed to reach significance, *p*’s > 0.45.

Additional analyses focused on response times on correct trials. To determine if there were any differences in saliency/discrimination across the different oddballs, we submitted unimodal response times to a 2 (modality: auditory vs. visual) × 4 (oddball: bear, cat, elephant, frog) repeated-measures ANOVA. While participants were faster at responding to visual stimuli (mean = 459 ms, *SE* = 13.52) than the auditory stimuli (mean = 544 ms, *SE* = 34.55), *F*(1,27) = 5.58, *p* = 0.026, *η*_p_^2^ = 0.17, BF10 = 1.49 × 10^5^, there were no differences in response times across any of the oddballs and response times to the different oddballs did not interact with stimulus modality, *p*’s > 0.70; therefore, we averaged across the oddball types (see Figure 9B for response times across modality and presentation mode).

Log-transformed response times on correct trials were submitted to a 2 (modality: auditory vs. visual) × 2 (presentation mode: unimodal vs. cross-modal) repeated-measures ANOVA. The analysis revealed a main effect of presentation mode, *F*(1,27) = 72.67, *p* < 0.001, *η*_p_^2^ = 0.73, BF10 = 9.95 × 10^7^, with response times in the cross-modal conditions being 149 ms slower than in the unimodal conditions. The analysis also revealed a modality × presentation interaction, *F*(1,27) = 12.74, *p* < 0.001, *η*_p_^2^ = 0.33, BF10 = 2.05 × 10^7^. As in Experiment 1, compared to the unimodal baselines, cross-modal presentation slowed visual responses (227-ms slowdown) more than auditory responses (71-ms slowdown). The effect of modality failed to reach significance, *p* = 0.67.

**FIGURE 10:**
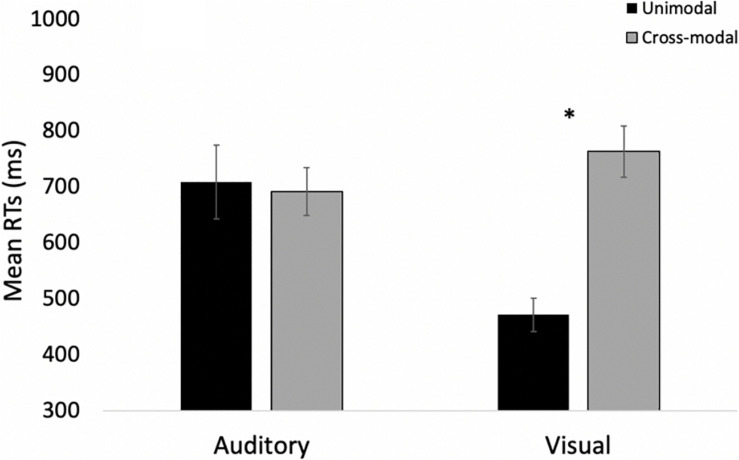
Mean response times across trial types and conditions in Experiment 3 for only those participants inhibiting responses to double oddballs. Error bars denote standard errors, and “*” denotes cross-modal means differ from unimodal means, *p*’s < 0.001.

Many participants in Experiment 3 struggled to inhibit their responses on double oddballs. The proportion of correct responses (i.e., no response) for the group was 51%, with 16 out of 28 participants false alarming more than 50% of the time. It is important to further analyze these trials because it is possible that these data replicate the overall pattern of Experiment 1 because many participants in the current experiment performed the same task as Experiment 1 and did not try to inhibit their responses on double oddballs. To address this concern, we examined only participants who withheld their responses on at least 11 of the 16 double oddball trials. Overall, 10 participants met this criterion (84% correct on double oddball trials), and even when analyzing this subset of participants who accurately inhibited their responses, data replicate Experiment 1. A 2 (modality: auditory vs. visual) × 2 (presentation mode: unimodal vs. cross-modal) repeated-measures ANOVA revealed a main effect of presentation, *F*(1,9) = 12.08, *p* = 0.007, *η*_p_^2^ = 0.57, BF10 = 7.62, with cross-modal presentation slowing down response times by 137 ms. The analysis also revealed a modality × presentation interaction, *F*(1,9) = 9.33, *p* = 0.014, *η*_p_^2^ = 0.51, BF10 = 9.33. As can be seen in Figure 10, compared to the unimodal baselines, cross-modal presentation slowed visual responses (292-ms slowdown) more than auditory responses (17-ms speed-up). The effect of modality was not significant, *p* = 0.09.

#### Time-Locked HR Analyses

**FIGURE 11:**
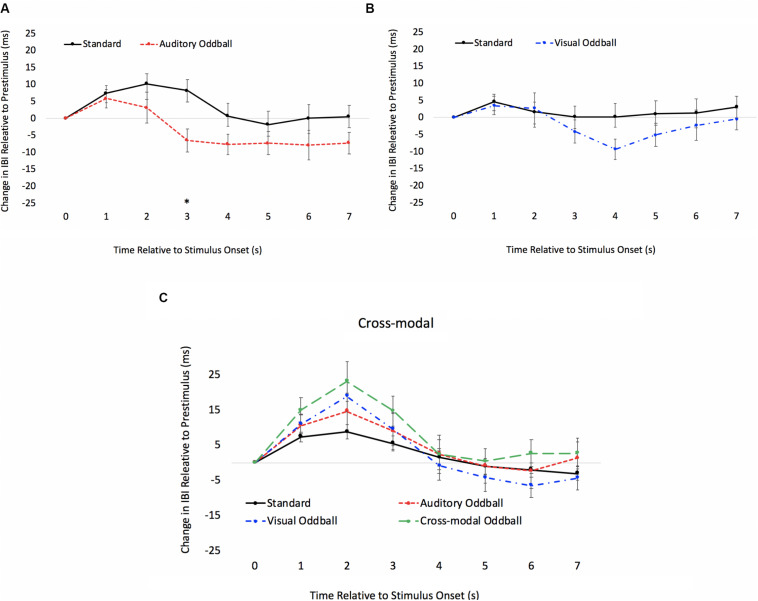
Cardiac responses in Experiment 3 across time in unimodal auditory **(A)**, unimodal visual **(B)**, and cross-modal conditions **(C)**. Error bars denote standard errors.

The HR waveforms for unimodal auditory, unimodal visual, and cross-modal conditions are presented in Figures 11A–C, respectively. As can be seen in the figures, cardiac discrimination across all three conditions was not as strong as in previous experiments, with no discrimination in the visual or cross-modal conditions. However, consistent with Experiments 1 and 2, cardiac responses to changing auditory and visual information did not corroborate behavioral data, which showed slower discrimination in the cross-modal condition.

#### Predictors of Modality Dominance

**FIGURE 12:**
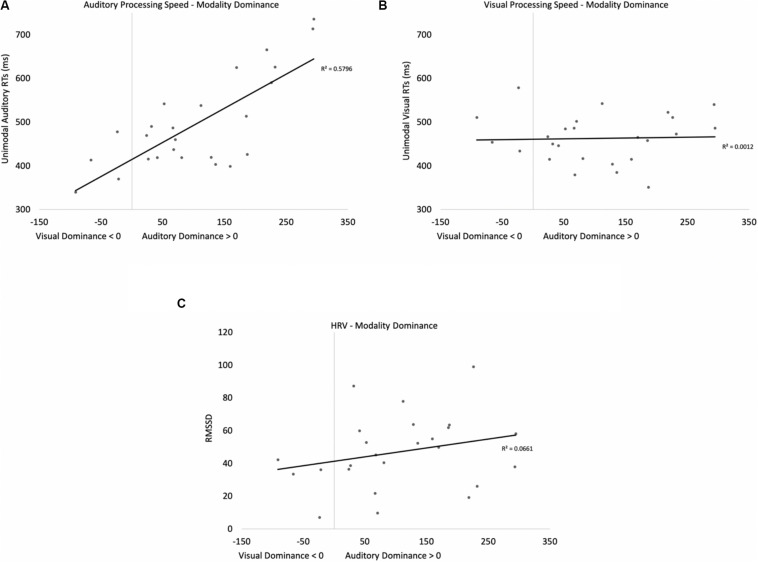
Scatterplots and effect sizes for unimodal auditory processing **(A)**, unimodal visual processing **(B)**, and HRV **(C)** with modality dominance (cost of cross-modal presentation on visual – cost of cross-modal presentation on auditory) in Experiment 3. Values greater than zero on the *x*-axis are consistent with auditory dominance.

As in previous experiments, we examined if HRV and processing speed could account for modality dominance (see Figure 12 for the bivariate scatterplots and effect sizes for the three predictor variables). Three participants were removed because their data were greater than two standard deviations above the mean. Eighty-six percent of the participants exhibited greater costs on visual processing (auditory dominance). Heart rate variability (RMSSD), unimodal auditory response times, and unimodal visual response times served as predictor variables in a stepwise regression to determine which variables account for modality dominance. While auditory processing speed accounted for 58% of the variance in modality dominance (slower auditory processing was associated with stronger auditory dominance), visual processing speed and HRV accounted for 1% and 7% of the variance, respectively. As in Experiment 1, the regression yielded two significant models. In the first model, auditory processing speed (*b* = 0.76) significantly predicted modality dominance, *F*(1,23) = 31.71, *p* < 0.001, *R*^2^ = 0.58, BF10 = 1501.07. In the second model, auditory processing speed (*b* = 1.00, *p* < 0.001) and visual processing speed (*b* = −0.48, *p* < 0.001) both predicted modality dominance, *F*(2,22) = 33.11, *p* < 0.001, *R*^2^ = 0.87, BF10 = 5.82 × 10^4^, with auditory dominance being more pronounced in participants who were slower at processing the sounds or faster at processing the pictures. However, as in Experiment 1, caution is required when interpreting the unimodal visual response time data given its correlation with auditory response times, *r*(23) = 0.51, *p* <0.009. Heart rate variability was not correlated with auditory or visual processing speeds (*p*’s > 0.24), and it did not significantly account for variability in modality dominance effects, *p* = 0.22.

## Across Experiment Comparisons

The results of Experiments 1 and 2 suggest that requiring participants to make separate responses to auditory and visual oddballs (visual response bias) and/or increasing cognitive load was responsible for the different patterns of modality dominance across experiments. Experiment 3 increased cognitive load while requiring participants to make the same response to auditory and visual oddballs. While proportions correct on auditory and visual oddball trials were comparable across Experiments 1 and 3 (97% vs. 96%, respectively), cross-modal presentation slowed down responding more in Experiment 3 (mean = 227 ms) than in Experiment 1 (mean = 33 ms), *t*(64) = 7.84, *p* < 0.001, BF10 = 1.07 × 10^11^. This finding suggests that the response inhibition manipulation in Experiment 3 significantly added to the complexity of the task. Under the more challenging conditions, the findings are more consistent with auditory dominance, which suggests that requiring separate responses for auditory and visual information was responsible for eliciting visual dominance in Experiment 2.

## General Discussion

Participants in the three reported experiments were presented with unimodal and cross-modal oddball tasks where they had to quickly respond when either the auditory, visual, or both components changed. When participants in Experiment 1 made a single response to auditory and visual oddballs, auditory dominance was found with cross-modal presentation slowing down visual responses more than auditory responses. However, when participants in Experiment 2 had to make different responses to auditory and visual oddballs, the pattern reversed with visual stimuli decreasing auditory oddball detection and participants making more visual- than auditory-based errors, the signature pattern of visual dominance. The auditory-to-visual dominance reversal could stem from a visual response bias or from increased cognitive load. Experiment 3 increased cognitive load by requiring participants to inhibit responses on trials when both auditory and visual modalities changed, while at the same time, participants made a single response to auditory and visual oddballs. Under the more challenging, single-response condition in Experiment 3, auditory dominance was found with cross-modal presentation slowing down visual responses more than auditory responses.

While the behavioral data show patterns of modality dominance shifting across the different response conditions, the time-locked cardiac responses to oddballs provide a different picture. It was hypothesized that auditory dominance may stem from auditory stimuli disrupting encoding of the visual stimulus (Robinson and Sloutsky, 2010b). If auditory stimuli disrupt or delay visual encoding, then pairing a sound with the visual stimulus should slow down or delay visual response times as well as cardiac responses. The time-locked cardiac responses in Experiment 3 were not as robust as in Experiments 1 and 2; however, the first two experiments show that cardiac responses to oddballs were faster in the cross-modal conditions than in the unimodal conditions. More specifically, cardiac responses to standards and oddballs differed from each other earlier in the course of processing in the cross-modal condition, which should not have been the case if encoding was disrupted. This finding has important implications for potential mechanisms underlying modality dominance.

We also examined if auditory processing speed, visual processing speed, and HRV, a measure of attentional control, could predict modality dominance effects. Unimodal auditory and unimodal visual processing speed predicted auditory dominance in Experiments 1 and 3, with auditory dominance being more pronounced in individuals who were slower at responding to the sounds when presented unimodally (without pictures). Unimodal processing speed did not predict visual dominance in Experiment 2, and there was no evidence that HRV predicted modality dominance effects across any of the experiments.

### Mechanisms Underlying Modality Dominance

Over the last 40 years, there has been a considerable amount of research examining the Colavita visual dominance effect (Colavita, 1974; Sinnett et al., 2007; Ngo et al., 2010; Spence et al., 2012) and there is a clear pattern within the adult literature—when auditory and visual stimuli are simultaneously presented and participants have to quickly respond to this information, participants often respond to the visual stimulus and fail to report the auditory stimulus (but see Nava and Pavani, 2013; Hirst et al., 2018a for a different pattern in children). While Ngo et al. (2011) managed to reverse the effect (only under extreme conditions), it was not until recently that visual dominance has been consistently reversed (Dunifon et al., 2016; Robinson et al., 2016, 2018; Barnhart et al., 2018; Ciraolo et al., 2020). The primary goal of the current set of experiments was to develop a better understanding of modality dominance effects and factors that may account for different patterns across various paradigms.

The current findings contribute to previous research in several ways. First, while Colavita visual dominance effects are well documented, underlying mechanisms are poorly understood (Posner et al., 1976; Sinnett et al., 2007; Spence, 2009; Spence et al., 2012) perhaps as a result of a lack of a single mechanism that can account for the current findings. For example, while the modality appropriateness hypothesis (Welch and Warren, 1980) predicts that audition should dominate on temporal tasks and vision should dominate on spatial tasks, this account does not make strong predictions on change detection tasks where participants do not make spatial or temporal judgments. Our findings demonstrate either auditory (Experiments 1 and 3) or visual (Experiment 2) dominance, despite the experiments having identical spatial and temporal configurations.

It is also possible that visual dominance is the dominant pattern due to the inhibitory nature of connections between sensory systems (Desimone and Duncan, 1995; Duncan, 1996; Spence et al., 2012) with approximately 50% of the brain dedicated to the visual modality (Sereno et al., 1995) which could lead to vision dominating and/or modulating processing in other modalities. However, this account would predict visual dominance across all three reported experiments, rather than our demonstration of both dominance types.

Another account assumes that sensory modalities compete for attention, with auditory stimuli (due to their dynamic and transient nature) initially winning the competition (Robinson and Sloutsky, 2010b). However, this account would predict auditory dominance across all three experiments because the auditory stimuli should automatically engage attention and delay visual processing.

Finally, it is also possible that visual stimuli are less likely to engage attention than auditory stimuli, leading participants to develop an endogenous attentional bias toward vision to compensate for the poor alerting abilities of visual input (Posner et al., 1976). While this account predicts visual dominance in Experiment 2 where participants made modality-specific responses, it fails to explain the observed auditory dominance in Experiments 1 and 3.

Based on the behavioral data, we believe that two potential mechanisms are needed to fully account for the pattern of results. First, visual dominance effects disappeared when participants made the same response to auditory and visual oddballs. This finding is consistent with Posner et al. (1976) and might suggest that visual dominance effects stem from a visual response bias and occur later in the course of processing as participants are responding to visual information. Thus, visual dominance might reflect disruptions in responding, not encoding (see Spence, 2009 for a similar claim). In contrast, when participants in Experiments 1 and 3 used the same response button for auditory and visual oddballs, auditory dominance was found with cross-modal presentation slowing down visual processing. These effects may stem from auditory stimuli automatically engaging attention and attenuating or delaying visual processing (Robinson and Sloutsky, 2010b; see also Figure 1 for a processing model of this account).

The current findings are also consistent with research showing that cross-modal stimuli delay visual P300s in passive oddball tasks where no responses are made (Robinson et al., 2010) and delay the latency of first fixations to the visual stimulus (Dunifon et al., 2016; Barnhart et al., 2018). In both sets of studies, it is likely that auditory interference occurs during encoding, especially on tasks where no decisions/responses are made (Robinson et al., 2010). Interestingly, it is possible that auditory dominance effects might still be present but overlooked in situations where participants can develop a visual response bias. Support for this claim comes from a study using a similar methodology as the one reported in Experiment 2. In Ciraolo et al. (2020) participants completed unimodal auditory, unimodal visual, and cross-modal oddball tasks where the auditory and visual stimuli were unfamiliar, and the authors manipulated the relative timing of the auditory and visual stimuli. When participants made different responses to auditory and visual oddballs and auditory and visual stimuli shared the same stimulus onset and offset, adults made more visual-based errors on cross-modal trials, which is consistent with visual dominance. However, examination of response times showed a pattern consistent with auditory dominance. The current study found a similar pattern in Experiment 2. While participants also made more visual-based errors on cross-modal trials, cross-modal presentation slowed down visual processing more than auditory processing for 62% of the participants, a pattern that is consistent with auditory dominance.

The current study also expands on auditory dominance literature by examining what factors predict modality dominance. If auditory dominance reflects a race across sensory modalities, then the modality that is faster to engage attention should dominate the other modality. There is support for this claim when manipulating auditory and visual stimulus onset asynchronies (SOAs). When an auditory stimulus is presented before or at the same time as the visual stimulus, auditory dominance is found (Ciraolo et al., 2020). Ciraolo et al. (2020) were able to reverse this effect, but visual stimuli had to be presented 200 ms before auditory stimuli. The current study did not manipulate the SOA, but we found a different pattern of results when examining unimodal processing speed, with auditory dominance being more pronounced in participants who were slower at processing the sounds. This effect was especially pronounced in Experiment 3 where participants were approximately 200 ms faster at processing the unimodal visual stimuli compared to the unimodal sounds (Figure 10). Conceptualizing modality dominance as a race between sensory modalities, this condition should have resulted in visual dominance as visual stimuli should have won the race.

While it is unclear how to reconcile the current findings with Ciraolo et al. (2020) SOA manipulations, the data are consistent with two assumptions regarding a potential mechanism underlying modality dominance. The first assumption is that auditory stimuli are processed first during sustained attention, and the processing of the details of a visual stimulus does not begin until the auditory modality releases attention. If this is the case, then participants who are slower at processing the sounds should show the most pronounced auditory dominance because it will take longer for the auditory modality to release attention. This assumption was corroborated in both of the reported experiments showing auditory dominance (Experiments 1 and 3). Second, the auditory dominance account also predicts that sounds automatically grab attention in a bottom-up manner, and these effects are not under top-down attentional control. The current study found no relationship between HRV, a measure or top-down control, and modality dominance. This finding, in combination with previous research showing that attentional manipulations do not reverse modality dominance (Napolitano and Sloutsky, 2004; Robinson and Sloutsky, 2004; Sinnett et al., 2007; Ngo et al., 2010; Dunifon et al., 2016), suggest that factors other than endogenous attention may modulate dominance effects.

## Future Directions

In the following section, we focus on several factors that need to be addressed in future research. First, further examination of the relationship between changes in HR and behavioral responses is needed. One possible explanation for the dissociation between behavioral and psychophysiological responses is that HR might simply be an unreliable measure of sensory dominance; however, this seems unlikely given that all three experiments found similar patterns across the three different stimulus conditions. It is also possible that HR measures are simply more sensitive than behavioral responses (e.g., stronger signal). However, this explanation seems unlikely for two reasons. First, it would predict stronger effects, not a change in pattern (interference vs. facilitation). Second, it is also important to note that a more sensitive measure should result in increased sensitivity in all three conditions, including the unimodal baselines, whereas Experiments 1 and 2 show a stronger signal/faster discrimination in the cross-modal condition relative to the unimodal conditions. It is also possible that changes in HR are more sensitive to encoding/oddball detection, whereas response times reflect encoding and competition while participants are making a decision/response (i.e., response time data may overlook facilitation effects). Given the HR data and response demand manipulation effects, it is possible that both auditory and visual dominance stem from competition during later stages of processing (see also Robinson and Sloutsky, 2019, for a similar claim in young children); however, other methodologies such as Event Related Potentials (ERP) may be useful in future studies to better differentiate encoding from the decision/response phase.

Second, future research will also need to further examine the decision/response component, especially considering the HR data suggest that both auditory and visual interference may be occurring after encoding. To address this issue, it will be important to use variations of diffusion models (Ratcliff, 1978; Ratcliff and McKoon, 2008; Turner et al., 2017) to systematically examine how cross-modal presentation and response manipulations affect: (a) the quality of the incoming auditory and visual information, (b) the amount of evidence needed for participants to make a response, and (c) initial biases or sensitivities toward one modality over the other. However, diffusion models often use two-choice decision tasks to model accuracy and response time data. While Experiment 1 used a two-choice task, auditory and visual oddball detections were associated with the same responses; thus, additional research will either need to modify the task so it fits within a diffusion model framework or develop a model that can accommodate different response demands such as the ones employed in the current study.

Third, a potential mechanism underlying auditory dominance assumes that processing speed of the auditory modality should predict the latency of visual processing, whereas given the serial nature of processing (auditory processed first), it also predicts that visual processing speed should not predict interference of already processed auditory information. Data from Experiments 1 and 3 show that auditory processing speed predicted auditory dominance; however, regressions in both experiments show that visual processing speed also predicted modality dominance, albeit to a lesser extent. While future research is needed, this likely stems from multicollinearity and using highly correlated variables in a regression rather than from visual stimuli independently predicting modality dominance. For example, as can be seen in Figures 5, 12, visual processing speed accounted for only 3% and 0% of the variance in modality dominance.

Fourth, within the auditory dominance literature, the signature pattern of modality dominance is that cross-modal presentation attenuates processing in the non-dominant modality while having little or no cost on processing in the dominant modality (asymmetric costs). Asymmetric costs can be found across any possible sensory pairings (e.g., touch and vision, etc.), and as with complex auditory and visual interactions, modality dominance effects in other sensory modalities likely interact with the nature of the sensory systems, presentation mode, and contextual factors. Thus, while we believe that general distinctions such as differentiating encoding and responding, serial versus parallel processing, and examining asymmetric costs will be useful when examining modality dominance in other sensory modalities, more specific assumptions such as why a particular sense may automatically engage attention may not generalize to other sensory pairings.

Finally, several smaller issues will also need to be examined in upcoming studies. First, participants in all three experiments were consistently responding at near-ceiling accuracy levels on oddballs. This is unfortunate for two reasons. First, given the lack of range, the regressions with accuracy were not significant; thus, future research will need to increase task difficulty to determine what factors best account for decreased accuracy on modality dominance tasks. Second, given the lack of range in accuracy data, it was difficult to properly examine speed-accuracy tradeoffs. While future research is needed, comparisons with the unimodal baselines suggest that it is unlikely that a speed-accuracy explanation can account for the current findings. For example, relative to the unimodal visual baselines in Experiments 1 and 3, cross-modal presentation had a cost on both accuracy and response times (as opposed to being inversely related), which is consistent with modality dominance and not a speed-accuracy tradeoff. Finally, while the current study used different procedures to examine oddball detection, additional research will need to further examine different types of oddball identification tasks (i.e., not just detecting oddballs, but making fine discriminations between different types of auditory and visual oddballs). Based on previous research using different methodologies to examine finer discriminations within each sensory modality (Sinnett et al., 2007, 2008; Dunifon et al., 2016; Barnhart et al., 2018) it is likely that the current effects will generalize to oddball identification tasks.

## Conclusion

In summary, many tasks require the simultaneous processing of auditory and visual information, and research with adults over the last 40 years demonstrates that the visual modality often dominates processing in the auditory modality. The current study replicated visual dominance when participants made different responses to auditory and visual information; however, these effects reversed to auditory dominance when participants made the same response to auditory and visual information. Moreover, auditory dominance was best predicted by auditory processing speed with slower auditory processing resulting in stronger auditory dominance. While behavioral data point to cross-modal interference, cardiac data point to cross-modal facilitation. These findings shed light on tasks that hinge on the processing of cross-modal information while suggesting that modality dominance might be occurring late in the course of processing.

## Data Availability Statement

The datasets generated for this study are available on request to the corresponding author.

## Ethics Statement

The studies involving human participants were reviewed and approved by the Behavioral and Social Science Institutional Review Board at The Ohio State University. The patients/participants provided their written informed consent to participate in this study.

## Author Contributions

CR designed the study, programmed the experiment, analyzed the data, and wrote up the first draft of the manuscript. KC and JP analyzed the data and contributed to manuscript writing. SS designed the study and contributed to manuscript writing. All authors contributed to the article and approved the submitted version.

## Conflict of Interest

The authors declare that the research was conducted in the absence of any commercial or financial relationships that could be construed as a potential conflict of interest.

## Footnotes

1 We also used regressions to examine accuracy data; however, participants were at near ceiling performance in all three experiments (limited range), and none of the regressions reached significance.
